# Uncovering Neural Learning Dynamics Through Latent Mutual Information

**DOI:** 10.3390/e28010118

**Published:** 2026-01-19

**Authors:** Arianna Issitt, Alex Merino, Lamine Deen, Ryan T. White, Mackenzie J. Meni

**Affiliations:** NEural TransmissionS (NETS) Lab, Florida Institute of Technology, Melbourne, FL 32901, USA; aissitt2019@my.fit.edu (A.I.); amerino2022@my.fit.edu (A.M.); ldeen2016@my.fit.edu (L.D.); mackenzie.meni@gmail.com (M.J.M.)

**Keywords:** representation learning, mutual information, learning dynamics, channel specialization, information theory, HSIC regularization, interpretability, XAI, deep learning

## Abstract

We study how convolutional neural networks reorganize information during learning in natural image classification tasks by tracking mutual information (MI) between inputs, intermediate representations, and labels. Across VGG-16, ResNet-18, and ResNet-50, we find that label-relevant MI grows reliably with depth while input MI depends strongly on architecture and activation, indicating that “compression’’ is not a universal phenomenon. Within convolutional layers, label information becomes increasingly concentrated in a small subset of channels; inference-time knockouts, shuffles, and perturbations confirm that these high-MI channels are functionally necessary for accuracy. This behavior suggests a view of representation learning driven by selective concentration and decorrelation rather than global information reduction. Finally, we show that a simple dependence-aware regularizer based on the Hilbert–Schmidt Independence Criterion can encourage these same patterns during training, yielding small accuracy gains and consistently faster convergence.

## 1. Introduction

Modern neural networks can achieve high accuracy while revealing little about how they organize information internally. This hidden structure governs what a model can transfer, how it fails under distribution shift, and whether we can reliably interpret or intervene in its behavior. As these systems move into sensitive and high-stakes applications, understanding how they encode and transform information becomes a practical requirement rather than a theoretical curiosity.

To make this internal organization explicit, we use mutual information (MI) to quantify how strongly each representation depends on the input and how much label-relevant structure it encodes. Measuring MI at both the layer and channel levels reveals how task-relevant information accumulates with depth and how networks reshape input variability as they learn, often concentrating discriminative signal into a small subset of channels.

We track these quantities over the entire training trajectory rather than at isolated checkpoints. This dynamic view shows when label-informative structure emerges, when it stabilizes, and why input-information “compression’’ appears only under specific architectural and activation choices rather than as a universal training phenomenon.

The contributions of this work are:

1. Layer- and channel-level MI analysis. A unified MI framework for quantifying how input- and label-relevant information propagate through CNNs during training.
2. Inference-time MI patterns across architectures. Consistent growth of label MI with feature depth and activation-dependent behavior of input MI across VGG-16, ResNet-18, and ResNet-50.
3. Functional specialization of channels. We identify compact subsets of high-MI channels and demonstrate their necessity through knockouts, shuffles, and perturbations.
4. Regularization aligned with MI structure. An HSIC-based regularizer reinforces these MI patterns, reducing redundancy and improving both accuracy and convergence speed.

These contributions show that MI exposes stable and functionally meaningful structure in CNN representations under the studied training setting—structure that is otherwise invisible through standard evaluation and that can be shaped through explicit regularization.

Accordingly, this work characterizes reproducible information-allocation patterns within a controlled and widely studied vision setting, rather than asserting dataset-independent laws.

## 2. Related Work

Mutual information (MI) quantifies statistical dependence between variables, and has been widely used to study how neural networks encode structure. For random variables *X* and *Y* with joint density *f* and marginals $f_{X}$ and $f_{Y}$,

$$I\left(X;Y\right)=E\;\left[\log\frac{f(X,Y)}{f_{X}\left(X\right)f_{Y}\left(Y\right)}\right],$$

which is non-negative and vanishes only under independence. Within deep networks, MI provides a way to characterize the degree to which internal features retain input variability or capture label-relevant signal.

### 2.1. Estimating MI in Latent Spaces

High-dimensional MI is notoriously hard to estimate. Nearest-neighbor estimators such as KSG [1], variational methods such as MINE [2], and contrastive bounds such as InfoNCE [3] offer different tradeoffs in bias, variance, stability, and computational cost. Because these estimators often disagree in scale and occasionally in trend, many representation-focused studies emphasize comparative analyses within a single estimator family. Following this principle, we adopt a fixed histogram-based estimator and restrict attention to relative patterns across layers, channels, models, and training stages to emphasize changes in the representations themselves rather than differences in estimator behavior.

### 2.2. MI as a Learning Principle

Early information-theoretic formulations framed learning as maximizing retained information (InfoMax) [4,5], or conversely as trading off relevance and compression through the information bottleneck (IB) [6]. Empirical studies initially suggested a stereotyped “fitting then compression’’ pattern in I(X;T) during training [7]; however, subsequent work showed that this behavior is highly sensitive to activation functions, normalization layers, and choice of MI estimator [8]. More recent analyses have argued that compression is not a universal feature of training but emerges only in certain architectural or optimization regimes [9]. These debates highlight the need for measurement frameworks that emphasize internal consistency rather than absolute MI values.

### 2.3. Information-Guided Regularization

A parallel line of work uses information-theoretic criteria not only for analysis but as training signals that shape representation learning. Entropy-based regularizers have been shown to encourage desirable information flow patterns across layers, reduce redundancy, and improve convergence in dense and convolutional networks [10,11]. Such methods demonstrate that information structure can be steered during optimization rather than merely measured post hoc. Related ideas appear in mutual information-maximizing objectives such as InfoMax [4], MI-based feature selection [12], and dependence-promoting losses in representation learning [5]. Our approach aligns with this perspective; we analyze MI flow to identify characteristic patterns of specialization, then use HSIC, a stable and differentiable proxy for dependence, to gently bias training toward those structures.

While variational and contrastive MI estimators such as MINE and InfoNCE have become popular training time surrogates, they introduce additional instability due to critic parameterization, variance from negative sampling strategies, and scale inconsistency across batches. In contrast, HSIC provides a critic-free kernel-based dependence measure that is stable to estimate on mini-batches and differentiable without requiring density models or contrastive sampling. This makes HSIC a pragmatic regularizer in settings where MI objectives are desirable but direct MI estimation is computationally heavy or statistically volatile.

### 2.4. Representation Analysis Perspective

Prior analyses of neural representations emphasize that learned structure arises from interactions between architecture, data, and optimization rather than from universal information-theoretic laws. Here, we use MI as a diagnostic to quantify how information is distributed across layers and channels, allowing us to characterize the emergence of representational structure and channel-level specialization during training.

## 3. Methods

Our goal is to measure how CNNs redistribute input- and label-relevant information across depth during learning. We track the mutual information (MI) between inputs, intermediate activations, and labels across training epochs using a fixed histogram estimator applied uniformly across models and epochs. MI values are interpreted in relative terms within each model, enabling consistent comparisons of information flow and specialization over training.

### 3.1. Model Architectures

We study three standard CNN classifiers: VGG-16 [13], ResNet-18, and ResNet-50 [14]. For VGG-16, we retain the full convolutional backbone and replace the classifier head with two linear layers (4096 and 10 units) with a ReLU and 50% dropout on the first. ResNet-18 and ResNet-50 use their unmodified architectures.

MI is measured at well-defined activation sites. For VGG-16, we extract post-ReLU convolutional activations after every convolutional operation. For ResNets, each residual block provides two measurement points: (i) the output of the residual branch before skip-addition, and (ii) the post-addition output after the skip connection. These paired measurements allow us to separate changes introduced by the residual transformation from those due to residual aggregation.

### 3.2. Datasets and Training Setup

All experiments use the Imagenette2 dataset [15] with its standard training/validation split. Unless otherwise noted, training follows a consistent protocol: random resized crops and horizontal flips for augmentation, normalization matched to ImageNet statistics, and training from scratch using SGD with momentum 0.9, weight decay $5\times 10^{-4}$, and a batch size of 64. The initial learning rate is $10^{-3}$ with ReduceLROnPlateau on validation accuracy (patience 10) and early stopping after 25 epochs without improvement. Each randomized experiment is averaged over five independent seeds.

Scope of Empirical Evaluation.

All experiments in this work are conducted on Imagenette2, a curated subset of ImageNet that preserves natural-image statistics while enabling dense information-theoretic measurements across training. Accordingly, our conclusions concern relative mutual information trends, channel-level specialization, and dependence structure in CNNs trained for natural image classification under ImageNet-like conditions. We do not claim universality across datasets or modalities, and treat generalization to larger datasets (e.g., full ImageNet) or other domains as an important direction for future research.

### 3.3. Histogram-Based MI Estimator

We estimate mutual information using a fixed histogram-based estimator applied to paired samples $\left(a_{i},b_{i}\right)$ collected from the validation set. Each variable is discretized into *B* uniform bins, yielding empirical joint and marginal densities

$$\hat{f}\left(a,b\right)=\frac{n_{ab}}{N},\;{\hat{f}}_{X}\left(a\right)=\sum_{b}\hat{f}\left(a,b\right),\;{\hat{f}}_{Y}\left(b\right)=\sum_{a}\hat{f}\left(a,b\right),$$

from which the MI estimate is

$$\hat{I}\left(A;B\right)=\sum_{a,b}\hat{f}\left(a,b\right)\;\log\;\left(\frac{\hat{f}\left(a,b\right)}{{\hat{f}}_{X}\left(a\right)\;{\hat{f}}_{Y}\left(b\right)}\right).$$

Histogram-based estimators are known to degrade in high-dimensional settings due to exponential growth in bin counts and sample requirements, so we do not apply MI estimation directly to high-dimensional activation tensors; instead, activation tensors are reduced by global average pooling, providing a stable summary representation with low estimator variance while preserving relative trends across layers.

Because the estimator, binning strategy, and preprocessing are held fixed for all layers, channels, architectures, and training epochs, the resulting MI values serve as consistent relative comparisons of how information redistributes within a model over the course of training.

### 3.4. Representation-Level MI Measurement

Let $C^{\left(\ell \right)}\in R^{H_{\ell }\times W_{\ell }\times D_{\ell }}$ be the activation tensor at layer *ℓ* and let $C_{c}^{\left(\ell \right)}$ denote channel *c*. For each checkpoint, we compute:

- $I(C^{\left(\ell \right)},Y)$ and $I(C_{c}^{\left(\ell \right)},Y)$: dependence between layer or channel activations and class labels.
- $I(X,C^{\left(\ell \right)})$ and $I(X,C_{c}^{\left(\ell \right)})$: retained dependence on the input.
- $I(C^{\left(\ell \right)},C^{\left(\ell^{\prime }\right)})$ and $I(C_{c}^{\left(\ell \right)},C_{c^{\prime }}^{\left(\ell^{\prime }\right)})$: inter-layer and inter-channel dependence.

All quantities use the same fixed histogram estimator (bin size 20) for consistency across layers, checkpoints, and architectures, enabling relative comparisons of how information is reallocated throughout training. A robustness analysis with respect to histogram bin count and sample size is provided in Appendix F.

Next, we introduce a training objective to encourage ideal dependency patterns among features and labels.

### 3.5. Hilbert–Schmidt Independence Criterion (HSIC) and Training Objective

MI offers a principled way to study dependence between neural variables, but direct MI estimation is difficult to use as a training signal; estimators rely on computationally expensive density estimation, nearest neighbors, or learned critics that are sensitive to dimensionality and introduce bias. The Hilbert–Schmidt Independence Criterion (HSIC) [16,17] provides a related kernel-based dependence measure that avoids density estimation.

Given paired samples ${\left\{\left(x_{i},y_{i}\right)\right\}}_{i=1}^{n}$ and kernels *k* and *ℓ*, the empirical estimate is

$$\hat{\mathrm{HSIC}}\left(X,Y\right)=\frac{1}{{\left(n-1\right)}^{2}}\mathrm{tr}\left(K_{c}L_{c}\right),$$

where *K* and *L* are the Gram matrices under *k* and *ℓ*, while $K_{c}$ and $L_{c}$ are their centered forms. HSIC equals zero only when *X* and *Y* are independent in the RKHS sense. As with MI, it captures both linear and nonlinear dependence; however, it is stable to estimate within a mini-batch and differentiable with respect to network parameters, making it suitable as a training loss.

Motivated by the representational patterns analyzed in later sections, we incorporate HSIC as a dependence-regularization term that encourages intermediate features to align more cleanly with class structure. During training, for each convolutional layer *ℓ*, we compute HSIC between the pooled activations $Z^{\left(\ell \right)}\;\in \;R^{B\times C_{\ell }}$ and the mini-batch labels, using an RBF kernel for features and a delta kernel for labels. The total loss is

$$L_{\mathrm{total}}=L_{\mathrm{CE}}\;+\;\alpha \sum_{\ell }\lambda_{\ell }\;\hat{\mathrm{HSIC}}\;\left(Z^{\left(\ell \right)},Y\right),$$

where α controls the strength of dependence regularization. This formulation allows the architecture training to favor the types of effective representational structures identified in our analysis.

In practice, HSIC introduces two tunable components: the global scale α controlling the overall strength of dependence regularization, and per-layer hyperparameters $\lambda_{\ell }$ determining how strongly each layer is encouraged to align with labels. We choose small values (α=0.01, $\lambda_{\ell }=10^{-3}$) to avoid overwhelming the cross-entropy gradient; Section 4.3.2 evaluates the sensitivity of the method to these hyperparameters. Computationally, HSIC adds only two Gram matrix multiplications per layer per batch. Because Gram matrices use globally pooled activations, HSIC scales with channel count rather than spatial size, making the overhead modest even for large networks.

### 3.6. Experimental Design and Analysis Framework

Our experiments combine post hoc MI measurements, within-layer specialization metrics, and training time dynamics to form a unified view of how CNNs acquire and distribute label-relevant information.

*Post Hoc Information Patterns across Layers and Architectures.*

We first examine how trained models allocate information across layers by computing input-to-layer MI $I(X;C^{\left(\ell \right)})$, layer-to-label MI $I(C^{\left(\ell \right)};Y)$, and inter-layer MI $I(C^{\left(\ell \right)};C^{\left(\ell^{\prime }\right)})$ for VGG-16 and ResNet-18. Comparing trained CNNs with their randomly initialized counterparts isolates structure produced purely through optimization and reveals global tendencies toward feature disentanglement, compression, hierarchical label encoding, and architecture-dependent retention of input variability.

*Channel-Level Specialization and Information Concentration.*

To quantify how label information is distributed within a layer, we compute the per-channel label MI $m_{c}=I\left(C_{c};Y\right)$ and form a probability distribution ${\hat{m}}_{c}=m_{c}/\sum_{j=1}^{C}m_{j}$. Let $p=({\hat{m}}_{1},\ldots ,{\hat{m}}_{C})$ denote the probability vector. $p_{\left(1\right)}\leq \cdots \leq p_{\left(C\right)}$ the ascending sort of *p*, and $p_{\left[1\right]}\geq \cdots \geq p_{\left[C\right]}$ its descending sort. We summarize within-layer structure using three standard concentration metrics: the Gini coefficient, Top-k% MI share, and normalized entropy:

$$\begin{array}{cccc} G(p) & =\frac{1}{C}\sum_{i=1}^{C}\left(2i-C-1\right)\;p_{\left(i\right)},\;\mathrm{Top}-k\%\left(p\right) & =\sum_{i=1}^{\left\lfloor kC/100\right\rfloor }p_{\left[i\right]},\;H_{\mathrm{norm}}\left(p\right) & =-\frac{1}{\log C}\sum_{i=1}^{C}p_{i}\log p_{i}. \end{array}$$

*Units.*

MI is reported in nats throughout, consistent with our use of natural logarithms. By contrast, the normalized entropy $H_{\mathrm{norm}}\left(p\right)=-\frac{1}{\log C}\sum_{i=1}^{C}p_{i}\log p_{i}$ is dimensionless since normalization by logC cancels the logarithmic information units.

We summarize within-layer structure using the Gini coefficient, which measures inequality in the MI distribution, the Top-*k*% MI share, which captures how much MI is concentrated in the most informative channels, and the normalized entropy, which quantifies the overall uniformity of MI across channels. These metrics quantify the extent to which training produces compact subsets of label-informative channels relative to random baselines.

*Training Time Information Dynamics and Regularization Effects.*

Representational structure develops gradually during learning; thus, we track each layer’s information trajectory over training. For each layer, we monitor $I(X;C^{\left(\ell \right)})$ across epochs to see when input structure is preserved, when it is discarded, and when label-relevant structure begins to dominate; for readability, we use the same notation to refer to the analogous layer–label and inter-layer quantities. These trajectories reveal when specialization emerges and whether it stabilizes or continues to drift. To test whether such dynamics can be shaped rather than simply observed, we introduce an HSIC-based regularizer and measure its effect on the timing and strength of label alignment, the concentration of information within channels, and overall accuracy. This allows us to evaluate whether promoting label dependence and reducing redundancy leads to more structured representations and faster convergence.

This methodology provides a unified approach to quantifying how CNNs encode, allocate, and refine information. By combining MI estimation, concentration metrics, and kernel-based dependence measures under a consistent validation protocol, we isolate representational changes arising from training rather than estimator or data artifacts.

## 4. Results

Our measurements reveal how CNNs rearrange information during learning: dependencies with inputs and labels shift predictably across depth, feature-label MI concentrates into small subsets of high-impact channels, and these structures emerge at distinct stages of training. These trends are consistent across architectures and can be strengthened through HSIC regularization, indicating that representation formation follows a selective, dependence-driven process.

### 4.1. Post Hoc MI Patterns Across Architectures

The experiments in this subsection will focus on patterns in MI we can measure post-training using only inference in different architectures.

*Input-Layer and Layer–Label MI Structure.*

**Figure 1:**
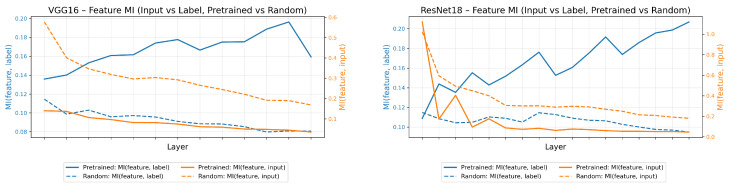
Mutual information (MI) between inputs, layer representations, and labels for VGG-16 and ResNet-18. Pretrained models (solid) vs. random initialization (dashed).

Figure 1 shows how MI redistributes across depth in trained versus randomly initialized networks. For both VGG-16 and ResNet-18, the ImageNet-trained models exhibit a clear rise in layer-label MI as depth increases: later representations encode markedly stronger dependence on the class labels, whereas early layers remain weakly informative. By contrast, random networks show uniformly low label MI across all depths.

The orange curves highlight the complementary pattern for input-layer MI. In trained models, dependence on the raw input decreases with depth, reflecting a transition from low-level features to more abstract task-aligned representations. Random networks again lack structure, maintaining uniformly higher input MI because their layers remain entangled with the input signal.

*Layer–Layer MI Structure.*

**Figure 2:**
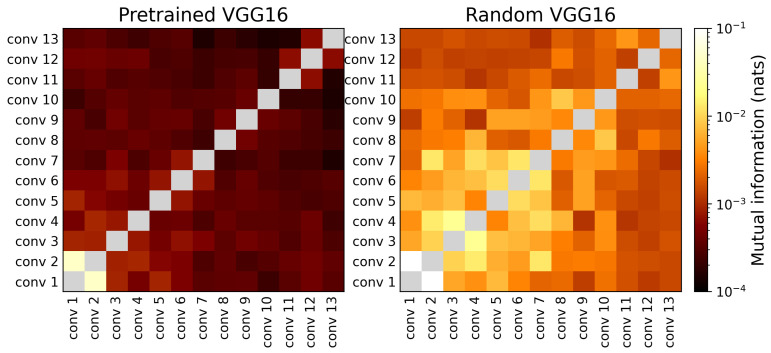
Pairwise MI between layer-wise representations, inputs, predictions, and labels for ImageNet-trained (**left**) and randomly initialized (**right**) VGG-16 classifiers.

Figure 2 visualizes the pairwise MI between intermediate VGG-16 representations. The ImageNet-trained network (left) shows a characteristic pattern: MI between distant layers is lower and MI decreases steadily with depth, indicating that successive transformations produce increasingly decorrelated feature spaces. The randomly initialized model (right) shows the opposite trend: significantly higher MI between many layers, reflecting redundant and entangled representations that have not yet specialized.

A notable exception in the trained network is the first convolutional pair: the MI for conv1–conv2 is roughly two orders of magnitude larger than for any other layer pair. These layers sit closest to the raw pixels and share highly overlapping receptive fields; thus, both effectively encode similar low-level statistics (edges, color blobs, local contrast) before later blocks reconfigure this front-end into more task-specific features. This pattern aligns with prior work using the PEEK [11] model visualization, where early convolutional filters appeared low-variance and visually unremarkable [18,19] yet were functionally critical and not safely prunable [20,21]. Entropy-based guidance results [10] showed that the first convolutional layers uniquely preserved most of the input entropy, unlike later layers.

These trends reveal that layers in well-trained CNNs carry strong label MI and weak input MI near the head, with weak layer–layer MI throughout. The model keeps what matters for classification, throws out what does not, and avoids redundant transformations. These patterns reveal a representation hierarchy that is selective, efficient, and structurally distinct from random baselines.

### 4.2. Channel-Level Specialization and MI Concentration

#### 4.2.1. Channel-Level MI Concentration

We measure how label information is distributed across channels by computing the per-channel MI $m_{c}=I\left(C_{c};Y\right)$ for each layer with $D_{\ell }$ filters and normalizing them across channels. We then assess the degree of MI concentration via the Gini coefficient (measuring inequality), Top-k% MI share (measuring share by highest-MI channels), and normalized entropy (measuring uniformity).

**Figure 3:**
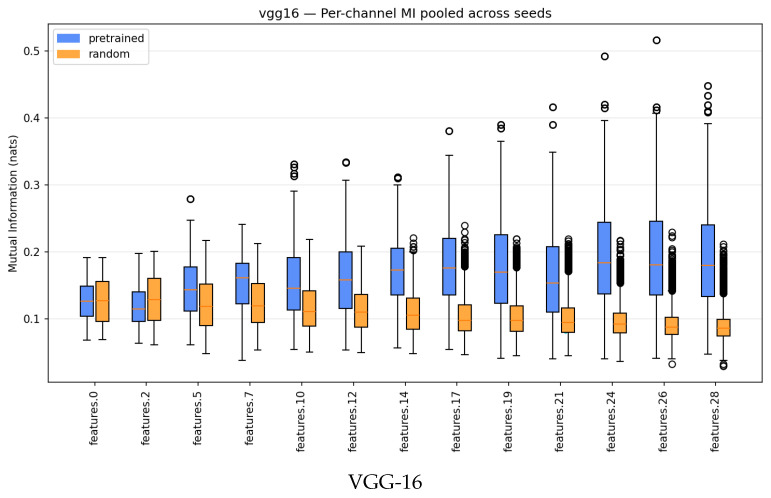

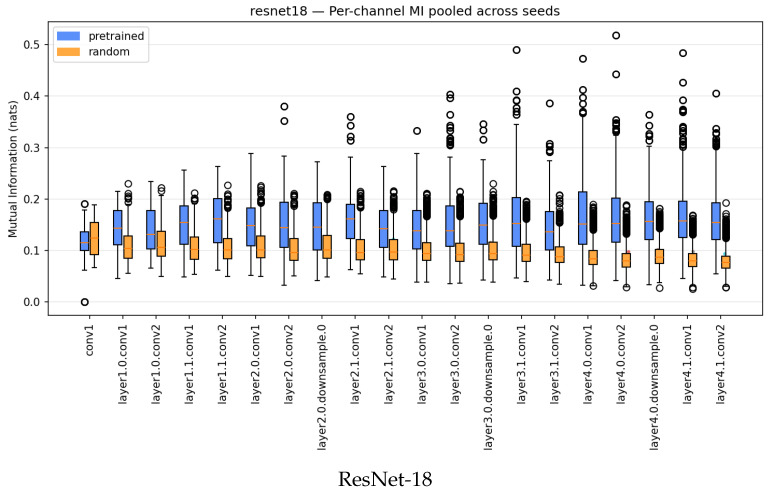
Per-channel MI boxplots across layers for pretrained vs. random networks. Trained models develop heavier upper tails and higher medians in later layers. Random models are flatter, with lower medians and tighter spreads.

**Figure 4:**
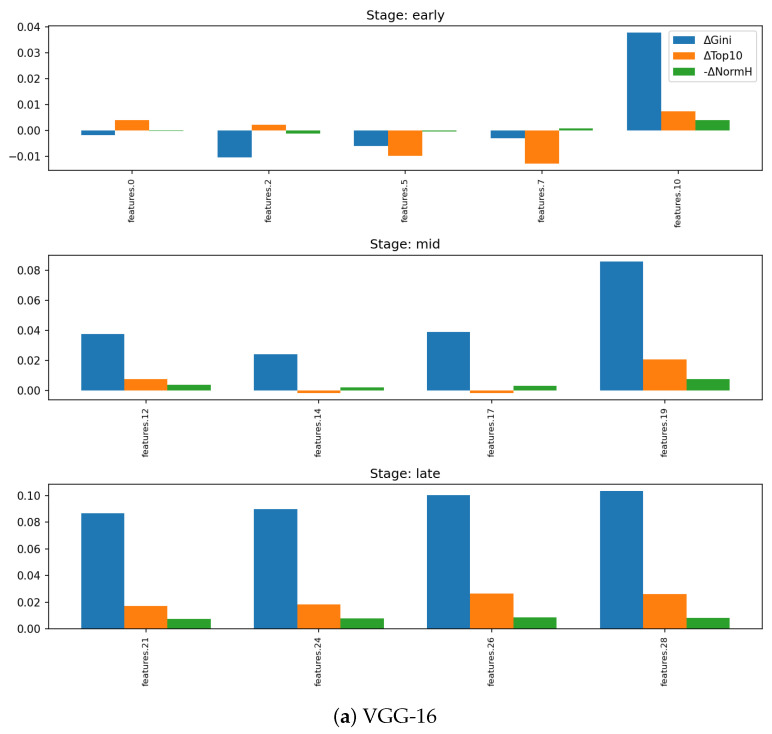

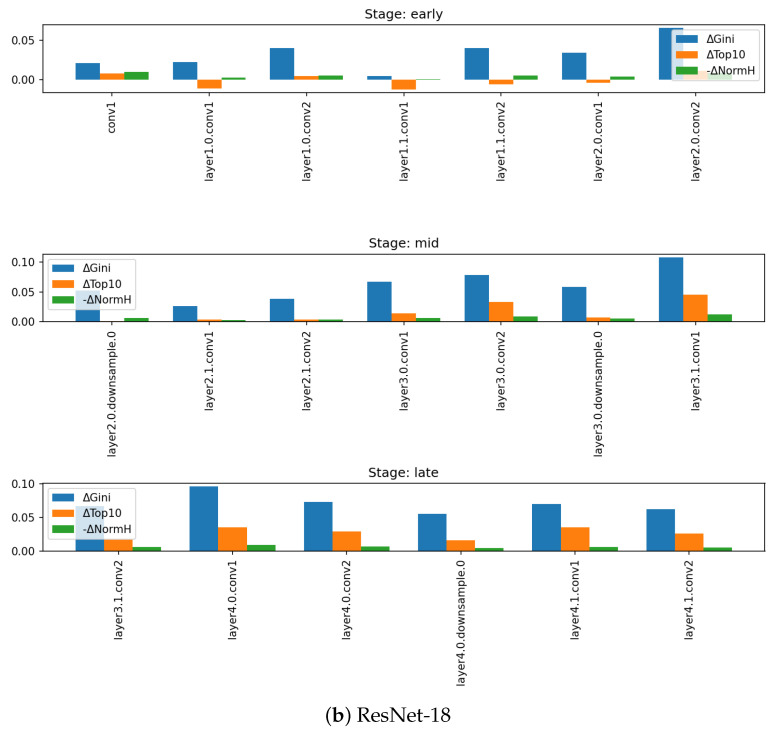
Per-layer deltas (trained–random) in sparsity metrics (Gini, Top-10% share, normalized entropy). Mid-to-late depths show the strongest increases in Gini and Top-10% share while normalized entropy remains stable, confirming that training concentrates label information into a subset of channels without collapsing overall diversity.

Across VGG-16 and ResNet-18, trained models show a clear shift toward more unequal MI distributions: Gini and Top-*k*% share rise consistently, especially in deeper layers, while normalized entropy decreases modestly. Random networks remain comparatively flat, with lower medians and tighter spreads across seeds. Figure 3 and Figure 4 show the emergence of heavier upper tails and larger positive training: random deltas in late layers, indicating that a small subset of channels becomes disproportionately label-informative.

These results highlight a robust pattern of within-layer specialization: training concentrates label information into a minority of channels while preserving overall diversity. This selective allocation provides a refined view of “compression” [6]; while layer-level MI may not contract strongly across layers with ReLU [8], the distribution of label dependence sharpens reliably.

#### 4.2.2. Functional Necessity of High-MI Channels

**Figure 5:**
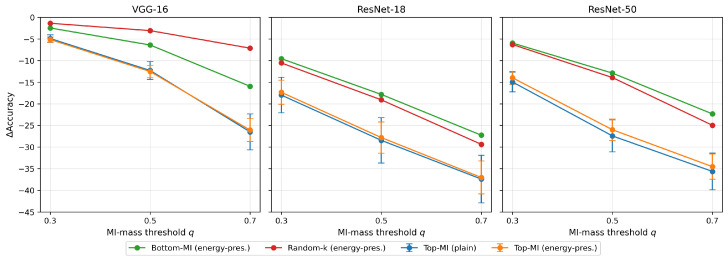
Inference time knockout experiments. For each model and MI-mass level *q*, removing the highest-MI channels produces substantially larger accuracy drops than removing random or low-MI channels, with similar effects under energy-preserving rescaling.

Having established that label information concentrates into a small subset of channels, we next test whether these channels are functionally necessary for inference. For each trained model, we rank channels within each layer by label MI $I(C_{c};Y)$ and perform knockout experiments that remove the top-MI channels until reaching cumulative MI-mass levels q∈{0.3,0.5,0.7}. These are compared against size-matched random and bottom-MI controls. All results are averaged over five seeds and shown in Figure 5.

We evaluate two variants: plain knockouts, which zero out the selected feature maps, and energy-preserving knockouts, which also rescale the remaining activations in the layer so that their $\ell_{2}$ norm matches the original pre-ablation magnitude. This controls for the possibility that accuracy drops might arise merely from reduced activation energy rather than from the removal of label-informative structure.

Top-MI knockouts consistently produce the largest accuracy drops across architectures and MI mass levels, showing that the channels carrying the most label information are also those most essential for prediction. Random and bottom-MI knockouts reduce accuracy far less, indicating that most channels contribute less to the decision. The energy-preserving variants, which restore the $\ell_{2}$ norm of the remaining activations, mirror the plain knockouts, confirming that the degradation is not an artifact of reduced feature magnitude. These results establish that high-MI channels encode task-critical structure rather than merely correlating with the labels.

A complementary analysis of residual blocks, including add-only MI concentration and skip-gain sweeps, is provided in Appendix A, where we show that skip connections primarily modulate how information is combined rather than which channels carry label-relevant signal.

Semantic and Architectural Localization.

**Figure 6:**
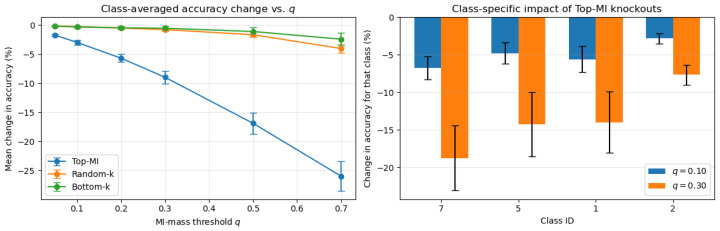
Class-targeted knockouts. Removing channels with the highest class-conditional MI causes much larger class-specific accuracy drops than removing random or low-MI channels. Effects grow with *q*.

We next test whether channel specialization aligns with semantic structure. For each class, we compute the class-conditional label MI and remove the smallest set of channels with cumulative MI mass that reaches a target level q∈0.05,0.10,0.30. Only predictions for that class are evaluated; performance on all other classes is left unchanged. Each targeted knockout is compared to size-matched random and low-MI controls (averaged over five seeds). Figure 6 shows a consistent pattern across all *q*: knocking out high-MI channels damages performance on the associated class far more than removing the same number of random or low-MI channels.

Across MI-mass levels, VGG-16 shows clear semantic dependence. At q=0.05, and typically only 6–8 channels, class accuracy drops by roughly 1.5–2% while random and low-MI controls remain near 0.1–0.3%. At q=0.10 (about 14–16 channels), targeted drops roughly double, with controls still minimal. By q=0.30, targeted effects become substantial; the most MI-dependent classes fall by 7–19%, while random controls at the same *q* remain around 0.5–1%. Full quantitative results, including per-class breakdowns and confidence intervals, are provided in Appendix B.

These findings show that high-MI channels form genuine class-specific support sets; each class depends on its own informative subset of “specialist” channels, and removing them selectively degrades that class while leaving others unaffected. Combined with the global knockout experiments, this demonstrates that MI ranking captures both overall importance and fine-grained semantic specialization in the network.

#### 4.2.3. Robustness and Redundancy

We assess how stable and redundant these specialists channels are, i.e., whether they can be transplanted between models or replaced by redundant channels.

Cross-Model Specialist Transplants.

To test whether high-MI channels are reusable across networks, we transplant the top-MI out-channels from a trained donor model into a shape-matched recipient of the same backbone trained with a different seed. Across VGG-16, ResNet-18, and ResNet-50, every transplant reduces validation accuracy, with losses increasing monotonically with the MI mass level *q*. Size-matched random and low-MI controls show comparable degradation, indicating that high-MI channels are not transferable in isolation; rather, their function depends on the representational context in which they emerge rather than reflecting universally reusable “feature specialists”. Full backbone-wise tables and confidence intervals appear in Appendix C.

Redundancy and Minimal MI Cover Analyses.

We further compare a minimal MI cover set (the smallest non-overlapping subset of channels needed to reach a target MI-mass) with redundant MI-matched sets that achieve the same mass using overlapping channels. Redundant sets buffer perturbations well at small *q*, but become harmful once a large fraction of the most informative channels is removed. This flip in behavior indicates that early-layer features are highly overlapping, while later layers carry specialized non-interchangeable information. Complete results for all architectures and MI mass levels are provided in Appendix D.

### 4.3. Training Time Information Dynamics and Regularization Effects

#### 4.3.1. Temporal Evolution of Information Flow

We analyze how I(X;T) and I(T;Y) evolve during training to assess when label structure emerges and whether the classic “fitting then compression” behavior holds. For each run, we summarize the change in input information with a single ΔI(X;T): the difference between initialization and the best-accuracy checkpoint, averaged over the final third of layers (where estimator noise is minimal). Negative values indicate net “compression”, while positive values indicate “expansion”.

**Figure 7:**
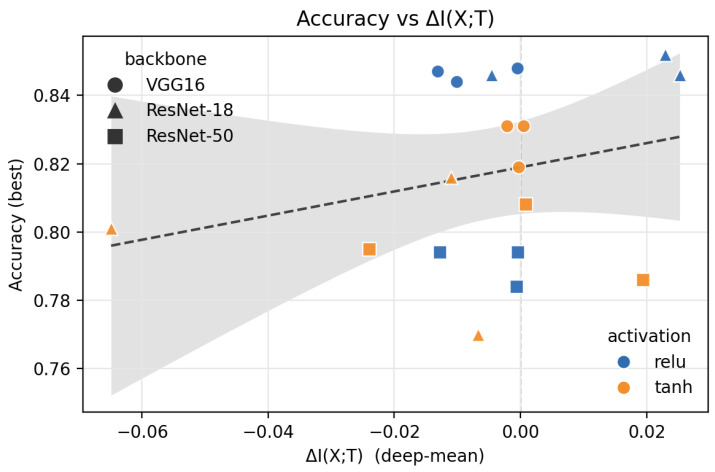
Accuracy vs. ΔI(X;T) (initialization → best, averaged over the final third of layers). The vertical line at x=0 separates compression from expansion. Compression is not universal; activation and architecture shape the trajectory.

Figure 7 shows accuracy versus ΔI(X;T) across VGG-16 and ResNet-18/50 using either ReLU or tanh activations (three seeds each). Points lie on both sides of zero with no downward trend; high performance does not require net compression, and many high-accuracy runs even expand input information. Activation choice matters more than accuracy; ReLU tilts toward expansion, while tanh produces a mix of compressive and expansive trajectories, consistent with prior findings that information plane behavior depends on activations rather than reflecting a universal training law [8].

**Figure 8:**
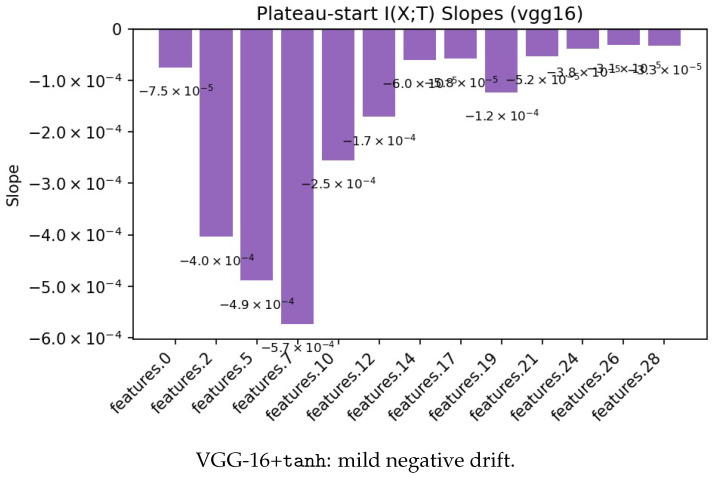

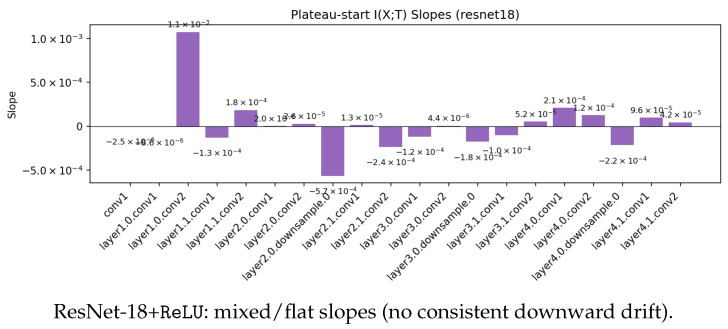
Post-plateau I(X;T) slopes (negative = downward drift). Any compression is small and architecture/activation specific.

To examine whether compression emerges after fitting, as sometimes suggested, we compute post-plateau slopes of I(X;T) using the data-driven plateau of I(T;Y) as the reference point. Representative examples in Figure 8 show two patterns: a mild downward drift in a tanh/VGG run, and near-zero or mixed slopes in a ReLU/ResNet run. Across all models and seeds, slopes are near 0 nats per epoch, indicating that when post-plateau compression appears, it is weak and entirely activation- and architecture-dependent.

Additional exploratory analysis of weight-level MI (Appendix E) did not reveal consistent trends within the methodology herein and diverges to separate research path. It is included for completeness.

#### 4.3.2. HSIC as Dependence-Based Regularization

We evaluate whether injecting a small HSIC term into the training objective improves optimization dynamics and final performance. HSIC serves as a differentiable proxy for mutual information, penalizing feature configurations that are statistically independent from the labels and rewarding those that express class-relevant structure. This makes it a natural counterpart to the representational patterns identified in our MI analysis.

We train two VGG-16 models on Imagenette2 with identical hyperparameters: one using standard cross-entropy (CE) and one augmenting CE with an HSIC regularizer applied to all convolutional layers (weights $\lambda_{\ell }\;=\;10^{-3}$, scale α=0.01). HSIC is computed batch-wise on globally pooled activations using RBF kernels for features and a delta kernel for labels. The comparison focuses on validation accuracy and validation loss over 100 epochs.

**Figure 9:**
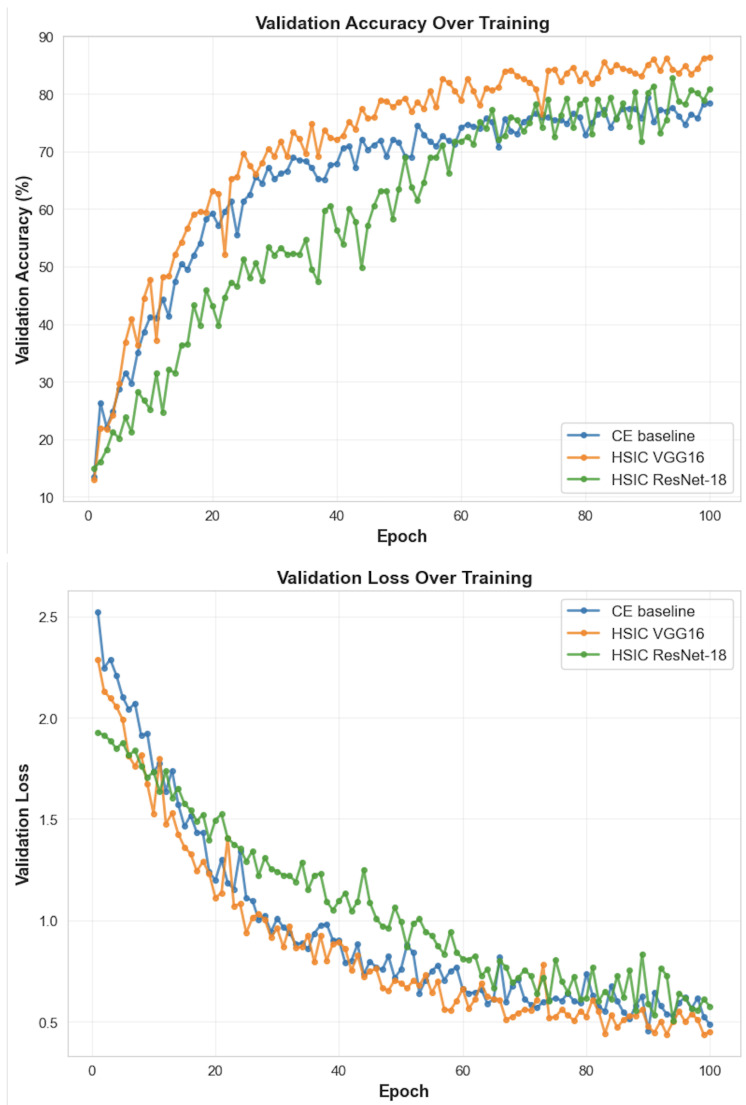
Validation performance for CE vs. HSIC-regularized training. HSIC yields faster convergence, higher peak accuracy, and smoother optimization behavior.

Figure 9 shows a consistent pattern across runs. The HSIC-augmented VGG-16 model reaches a higher validation accuracy (86.4%) than the cross-entropy baseline (79.4%). HSIC-augmentation of ResNet-18 provided a tiny accuracy boost (80.3%) as well. The validation loss mirrors this trend: HSIC drives a faster early decrease and reaches a lower final value, suggesting a more directed optimization path. Importantly, neither accuracy curves nor loss curves show signs of increased overfitting; both models remain slightly underfitted, and their generalization gaps are nearly identical.

These results indicate that the HSIC term acts not merely as a regularizer but as a dependence-guided training signal. By rewarding activations that align with class structure, HSIC accelerates the formation of discriminative features and reduces representational drift during learning. This complements our earlier MI findings: the dependence structure revealed in the post hoc analysis translates into tangible training time benefits when optimized directly. In short, HSIC provides an actionable way to impose the representational geometry that MI analysis suggests is desirable.

We also quantify compute overhead. The HSIC model runs 1.05× to 1.07× slower per epoch than the CE baseline due to the additional B×B Gram computations on pooled activations. This overhead remained consistent across tested batch size (64) and is minor relative to convolutional cost.

## 5. Conclusions

CNNs do not learn by uniformly compressing information. Instead, they form specialist channels consisting of a small subset of units that carry nearly all feature–label dependence, while the majority remain weakly informative or redundant. These specialists are functionally necessary, architecture-specific, and reproducible across runs, indicating that specialization is a structural property of learning rather than noise or estimator drift. These patterns arise consistently across architectures and training runs, and can be reinforced through a simple HSIC-based dependence regularizer that accelerates convergence and modestly improves accuracy.

Because Imagenette2 is drawn directly from ImageNet and preserves its natural-image statistics, supervision structure, and standard training pipelines, it provides a controlled proxy for ImageNet-scale vision tasks. While absolute mutual information values and optimization dynamics may shift with dataset scale, the qualitative patterns emphasized here (depthwise growth of label dependence, channel-level specialization, and dependence concentration) are tied to representational structure rather than dataset size. As such, Imagenette2 offers a practical setting for studying these mechanisms, even as direct validation on full ImageNet remains an important direction for future work.

More broadly, while the empirical results in this work are restricted to natural-image classification, the analysis framework itself is modality-agnostic. Whether similar MI dynamics and specialization patterns arise in other vision tasks or in non-visual domains such as language and audio remains an open question. Addressing this will require adaptation of MI estimation and representation analysis to domain-specific architectures and data structures, and is a focus of ongoing work.

Within this setting, these findings demonstrate that MI provides a stable and explanatory lens on representation formation and that the structures it reveals—specialization, sparsity, and dependence alignment—can be deliberately shaped during learning. 

## Figures and Tables

**Figure A1:**
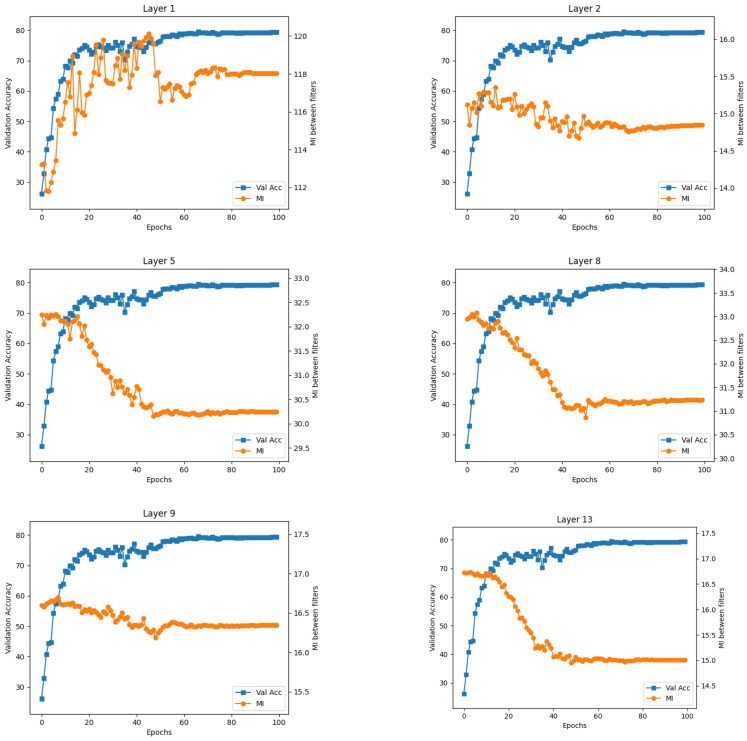
Accuracy and MI over 100 epochs. As the validation accuracy increases, the MI between filters follow suit for Layer 1. The MI calculated each epoch for layer 1 only stabilizes 15 epochs after the validation accuracy stabilizes. For Layers 5, 8, and 13, the MI largely decreases after epoch 16 and stabilizes around epoch 50, where the accuracy also stabilizes. Layers 2 and 9 decrease over the 100 epochs and exhibit no compression phase.

**Figure A2:**
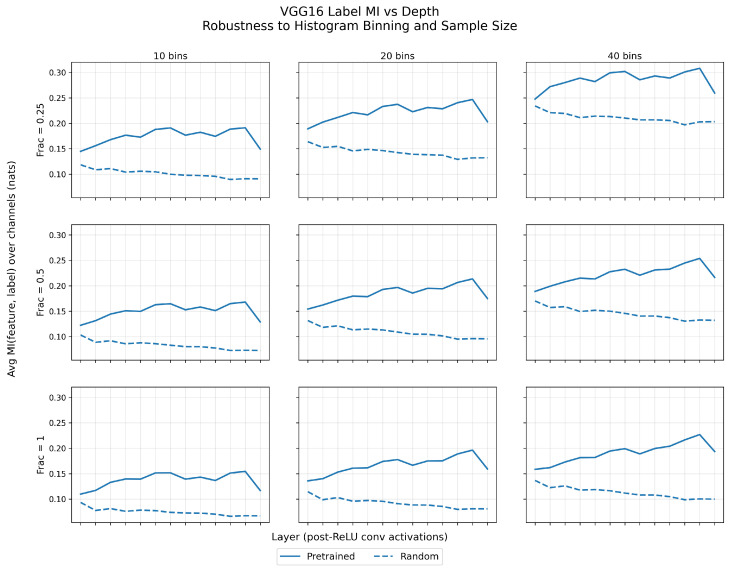
VGG-16 label MI vs. depth under varying histogram bin counts and dataset fractions.

## Data Availability

The Imagenette2 dataset used in this study is available on GitHub at https://github.com/fastai/imagenette (accessed on 17 October 2025), reference number [15].

## Appendix A. Skip Connections Modulate Within-Layer MI Concentration

This appendix expands the residual-network analysis by isolating how skip additions affect within-layer MI concentration and by quantifying the functional role of skip weights through gain sweeps. All results use the same MI estimator and validation protocol described in Section 3.

### Appendix A.1. Experiment A: Add-Only MI Concentration Analysis

This experiment isolates the effect of the skip-add operation by measuring how within-layer MI concentration changes from the residual branch output to the post-addition representation.

We instrument each residual block at three taps: pre (block input), sum (output of the residual branch before the skip add), and post (after the skip addition). Let $G(\hat{m})$ be the Gini coefficient computed on normalized per-channel label MI $\hat{m}=m_{c}/\sum_{j}m_{j}$. We decompose

$$\Delta_{\mathrm{branch}}=G_{\mathrm{sum}}-G_{\mathrm{pre}},\;\Delta_{\mathrm{add}}=G_{\mathrm{post}}-G_{\mathrm{sum}},$$

so $\Delta_{\mathrm{add}}$ isolates how the skip addition modifies MI concentration.

ResNet-18.

In the trained model, the skip addition slightly smooths concentration: $\Delta_{\mathrm{add}}G\approx -0.0066$ on average (identity skips: −0.0081, downsample skips: −0.0041). The random baseline instead increases concentration (identity: +0.0209, downsample: +0.0902). Depth-wise, trained blocks in the mid network reduce concentration (layer1.1: ≈−0.030, layer2.0: ≈−0.027), while the deepest blocks mildly increase it (layer4.0/4.1: ≈+0.027/+0.010).

ResNet-50.

Trained skip additions increase concentration throughout (identity: +0.038, downsample: +0.045), with the strongest effects in the final stage (layer4.2: ≈+0.217). The random baseline shows even larger positive spikes (identity: +0.059, downsample: +0.167).

**Table A1:** Skip-add effect on concentration. Values are aggregated over sites and seeds.

	ResNet-18		ResNet-50	
Condition × Skip	$\Delta_{\mathrm{add}}G$	$\Delta_{\mathrm{add}}\mathrm{NE}$	$\Delta_{\mathrm{add}}G$	$\Delta_{\mathrm{add}}\mathrm{NE}$
Trained × Identity	−0.0081	+0.0008	+0.0380	−0.0048
Trained × Downsample	−0.0041	+0.0010	+0.0448	−0.0072
Random × Identity	+0.0209	−0.0026	+0.0589	−0.0059
Random × Downsample	+0.0902	−0.0111	+0.1672	−0.0208

*Interpretation*

Skip additions have architecture-dependent effects: ResNet-18 uses the skip to smooth MI concentration, whereas ResNet-50 amplifies it. In both cases, random networks exaggerate these effects, confirming that trained models regulate the skip to maintain stable MI allocation.

### Appendix A.2. Experiment B: Global Skip-Gain Sweeps

This experiment probes the sensitivity of residual networks to global reweighting of skip connections by uniformly scaling all skip paths and measuring the resulting accuracy degradation.

We next vary the skip weight λ uniformly across all residual blocks:

post=λ·pre+sum.

This tests how sensitive the architecture is to global rebalancing of the skip and residual pathways.

**Table A2:** Global skip-gain sweep: accuracy change (pp) relative to trained model.

Backbone	λ=0	λ=0.5	λ=0.9	λ=1.0 (trained)	λ=1.3
ResNet-18	−74.7	−71.2	−7.4	0.0	−43.3
ResNet-50	−70.4	−71.0	−51.2	0.0	−60.2

Globally weakening or strengthening the skip severely harms accuracy. The network depends on a finely tuned skip–residual balance; global perturbations break this equilibrium.

### Appendix A.3. Experiment C: Per-Block Skip-Gain Sweeps

This experiment evaluates the robustness of individual residual blocks by perturbing skip gains one block at a time while keeping the rest of the network unchanged.

We next vary λ *one block at a time*, leaving all other skips at their original weight.

**Table A3:** Per-site skip-gain sweep: mean accuracy drops (pp).

Backbone × Skip	λ=0	λ=0.5	λ=0.9	λ=1.3
ResNet-18 Identity	−44.4	−13.6	−0.38	−7.1
ResNet-18 Downsample	−26.5	−6.7	−0.32	−5.8
ResNet-50 Identity	−54.4	−21.2	−1.10	−9.8
ResNet-50 Downsample	−39.8	−11.6	−0.49	−7.6

Local perturbations near the trained λ are far less damaging than global ones. Residual blocks are individually robust; failures arise from coordinated mis-scaling.

### Appendix A.4. Experiment D: Skip-Gain Effects on MI Concentration

This experiment examines whether skip-gain perturbations meaningfully alter within-layer MI concentration, distinguishing functional disruption from information reallocation.

Finally, we evaluate how skip-gain perturbations modify concentration at the add tap.

**Table A4:** Mean concentration change across skip-gain sweep.

	ResNet-18		ResNet-50	
Skip Type	$\Delta G_{\mathrm{add}}$	$\Delta {\mathrm{NE}}_{\mathrm{add}}$	$\Delta G_{\mathrm{add}}$	$\Delta {\mathrm{NE}}_{\mathrm{add}}$
Identity	+0.0011	−0.0001	+0.0049	−0.0003
Downsample	−0.0000	+0.0000	+0.0107	−0.0004

Skip-gain sweeps induce only tiny MI concentration shifts. Large accuracy swings are not driven by MI reallocation; they stem from functional disruption of the skip–residual balance. Skip paths chiefly control how information is combined, rather than which channels carry label-relevant signal.

### Appendix A.5. Summary

Skip connections exert subtle but important effects on MI concentration. The skip-add operation modulates sparsity differently across ResNet-18 and ResNet-50; however, in both cases the trained networks maintain stable concentration while random ones distort it. Global skip-gain changes catastrophically damage accuracy, whereas per-block changes are mild, indicating that residual networks rely on a globally coordinated skip–residual ratio. These analyses reinforce that skip connections shape information aggregation far more than they redistribute channel-wise label dependence.

## Appendix B. Class-Targeted Knockout Tables

This appendix reports full quantitative results for the class-targeted channel ablation experiment. For each class, channels within each layer are ranked by class-conditional label mutual information. We then remove the smallest set of channels for which the cumulative MI mass reaches a threshold q∈{0.05,0.10,0.20,0.30,0.50,0.70} and measure the resulting change in accuracy for that class. Size-matched random-*k* and bottom-*k* controls are included. All results are averaged over five seeds.

**Table A5:** Mean class-wise accuracy drop (in percentage points) for class-targeted knockouts across all classes and seeds. Negative values indicate reduced accuracy.

Model	*q*	Top-MI	Random-*k*	Bottom-*k*	$\bar{k}$
VGG-16	0.05	−1.74 [−1.98, −1.50]	−0.18 [−0.27, −0.11]	−0.18 [−0.28, −0.08]	7.4
	0.10	−2.99 [−3.38, −2.60]	−0.25 [−0.36, −0.14]	−0.33 [−0.48, −0.17]	15.6
	0.20	−5.70 [−6.37, −5.03]	−0.52 [−0.68, −0.35]	−0.47 [−0.72, −0.22]	34.9
	0.30	−8.99 [−10.06, −7.92]	−0.79 [−1.01, −0.56]	−0.55 [−0.93, −0.17]	58.0
	0.50	−16.90 [−18.71, −15.10]	−1.66 [−2.05, −1.27]	−1.11 [−1.77, −0.44]	117.3
	0.70	−25.99 [−28.55, −23.43]	−4.04 [−4.76, −3.32]	−2.43 [−3.53, −1.33]	200.7

**Table A6:** Largest class-specific accuracy drops for Top-MI knockouts at q=0.10. Values are percentage points, averaged over seeds.

Model	Class ID	Accuracy Drop	$\bar{k}$	Base Acc
VGG-16	7	−6.77 [−8.29, −5.25]	14.8	76.7
	1	−5.58 [−7.33, −3.84]	14.3	89.6
	5	−4.79 [−6.20, −3.39]	13.9	83.6
	2	−2.83 [−3.49, −2.16]	12.9	83.4
	3	−2.35 [−3.35, −1.35]	18.1	74.8

**Table A7:** Largest class-specific accuracy drops for Top-MI knockouts at q=0.30. Values are percentage points, averaged over seeds.

Model	Class ID	Accuracy Drop	$\bar{k}$	Base Acc
VGG-1	7	−18.75 [−23.06, −14.43]	53.5	76.7
	5	−14.25 [−18.53, −9.97]	50.0	83.6
	1	−13.99 [−18.06, −9.92]	55.8	89.6
	4	−9.47 [−12.46, −6.47]	56.2	92.4
	2	−7.67 [−9.00, −6.35]	48.9	83.4

These tables support the finding in the main text that Top-MI channels form class-specific support sets: removing them selectively impairs particular categories far more than size-matched random or low-MI controls. The number of channels removed grows with *q*, but the relative pattern remains the same: Top-MI knockouts consistently cause several-fold larger class-wise accuracy losses than either control condition.

## Appendix C. Cross-Model Specialist Transplants

This appendix provides the full tables and layer-wise results for the cross-model “specialist transplant’’ experiments. For each backbone, channels ranked by label MI in a trained donor network were transplanted into a shape-compatible recipient trained with a different seed. Accuracy deltas, per-layer effects, and control comparisons (random-*k*, bottom-*k*) are reported below. These results support the main text’s conclusion that high-MI channels are not freely reusable across networks without reoptimization.

## Appendix D. Redundancy vs. Minimal MI-Cover Analyses

This appendix contains full results for the redundancy experiments. We compare ablations of (i) minimal MI-cover sets (the smallest non-overlapping subset of channels needed to reach a target MI mass) and (ii) redundant sets that achieve the same MI mass with overlapping channels. The tables report accuracy deltas for both sets along with paired differences across VGG-16, ResNet-18, and ResNet-50. These results complement the finding in the main text that redundancy buffers performance at low MI mass but that this reverses once the high-MI tail is removed.

## Appendix E. Exploratory Analysis: Within-Layer Weight MI

For completeness, we computed MI between convolutional filters within individual layers (using histogram-based estimates on flattened kernels) to probe whether weight-space redundancy evolves during training. The results were highly estimator-sensitive and layer-dependent, with substantial noise in early layers as well as weak and architecture-specific trends. Because these patterns do not meaningfully align with or alter the activation-level MI results presented in the main text, we include this analysis only for transparency and do not draw strong conclusions. Figures and per-layer trajectories appear in Figure A1 and Table A8.

**Table A8:** Epoch when compression phase begins within each layer.

Layer Number	2	3	4	5	6	7	8	10	11	12	13
**Epoch**	65	8	24	20	22	17	17	23	18	15	15

The lack of consistent structure in weight-level MI indicates that MI between learned weights does not reliably track functional organization under the present experimental conditions. This suggests that weight-space MI is not inherently informative but may depend on additional constraints or regimes not explored here, such as explicit sparsity, stronger structural regularization, or late-stage fine-tuning where weight roles have stabilized. More generally, extracting meaningful information-theoretic structure from weight space may require estimators or representations better aligned with weight geometry. We leave a systematic investigation of these settings to future work.

## Appendix F. Robustness of Histogram-Based MI Estimation

Because histogram-based MI estimators require design choices such as binning resolution and sample size, it is important to verify that the conclusions drawn in the main text do not depend sensitively on these choices.

To test robustness to these choices, we computed all VGG-16 MI curves under systematic variations in both histogram bin count and effective dataset size. MI between pooled post-ReLU convolutional activations and the class label was estimated using three bin counts (10, 20, and 40) and three random subsamples of the validation set (25%, 50%, and 100%) while holding the network, preprocessing, and aggregation procedure fixed.

The resulting MI curves are shown in Figure A2 arranged by dataset fraction (rows) and histogram bin count (columns), with shared axes across all subplots. While the absolute MI values vary with estimator resolution and sample size, the curves exhibit the same depth-wise shape across all settings. In particular, the label-dependent information increases monotonically with depth for the pretrained network, remains substantially lower for the randomly initialized baseline, and preserves consistent relative ordering across layers. These observations confirm that the conclusions in the main text rely on stable relative structure (i.e., the form of the MI curves) rather than on the absolute magnitude of the MI estimates.
